# New Phoenix criteria for pediatric sepsis and septic shock: the strengths and the future of a comprehensive perspective

**DOI:** 10.62675/2965-2774.20240058-en

**Published:** 2024-07-01

**Authors:** Vanessa Soares Lanziotti, Andrea Ventura, Saraswati Kache, Jaime Fernández-Sarmiento

**Affiliations:** 1 Pediatric Intensive Care Unit and Research and Education Division Instituto de Puericultura e Pediatria Martagão Gesteira Rio de Janeiro RJ Brazil Pediatric Intensive Care Unit and Research and Education Division, Instituto de Puericultura e Pediatria Martagão Gesteira - Rio de Janeiro (RJ), Brazil.; 2 Department of Pediatrics Universidade de São Paulo São Paulo SP Brazil Department of Pediatrics, Universidade de São Paulo - São Paulo (SP), Brazil.; 3 Division of Critical Care Medicine Department of Pediatrics Lucile Packard Children’s Hospital Stanford California United States Division of Critical Care Medicine, Department of Pediatrics, Lucile Packard Children’s Hospital Stanford - California, United States.; 4 Fundación Cardioinfantil Instituto de Cardiologia Universidad de la Sabana Bogotá Colombia Department of Critical Care Medicine and Pediatrics, Fundación Cardioinfantil, Instituto de Cardiologia, Universidad de la Sabana - Bogotá, Colombia.

**Figure 1:**
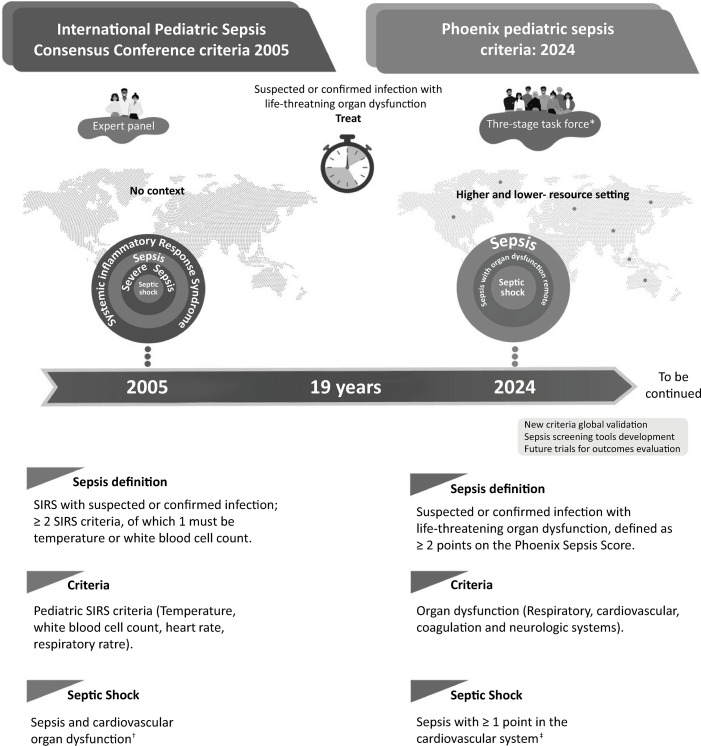
Comparison of the International Pediatric Sepsis Consensus Conference criteria with the Phoenix Pediatric Sepsis criteria in terms of objectives, definitions, criteria, classification and methodology. * International survey, systematic review/meta-analysis and a cohort study; † The International Pediatric Sepsis Consensus Conference Criteria definition of cardiovascular organ dysfunction is as follows: 40mL/kg or more isotonic fluid in 1 hour, hypotension or a need for vasoactive fluid or at least 2 of the following: unexplained metabolic acidosis, arterial lactate > 2 times the upper limit of normal, oliguria, a prolonged capillary refill time, or a core-to-peripheral temperature gap; ‡ The Phoenix definition of cardiovascular organ dysfunction includes severe hypotension for age, a venous or arterial blood lactate value of more than 5mmol/L (> 45.05mg/dL), or a need for vasoactive medication. SIRS - systemic inflammatory response syndrome.

In Greek mythology, the phoenix bird symbolizes life that overcomes death and the strength that accompanies transformation. Therefore, Phoenix is an appropriate name for the new Pediatric Sepsis Score owing to both the mythological reference and the location where it was first presented (Society of Critical Care Medicine – SCCM - Conference in Phoenix, Arizona).^(1)^The Phoenix Pediatric Sepsis (PPS) criteria for sepsis and septic shock are intended to identify children (1 month to <18 years) with life-threatening organ dysfunction due to infection, and the score was developed based on more than three million pediatric electronic health encounters,^(2)^which is a remarkable achievement considering pediatric and adult sepsis studies. The previous pediatric sepsis criteria were published in 2005 by the International Pediatric Sepsis Consensus Conference (IPSCC), and sepsis was defined as a suspected or confirmed infection in the presence of systemic inflammatory response syndrome (SIRS) (Figure 1).^(3)^Although these criteria are broadly used in daily practice, limitations to this definition have been identified since its inception.^(4)^Specific limitations of concern include a lack of consideration of a global context, leading to challenges in the applicabiblity of these criteria in limited-resource settings where the highest sepsis burden lies; variability in application at the bedside, which leads to delay in patient diagnosis; and the inability to identify the patients at greatest risk of poor outcomes.^(5)^

These IPSCC limitations have stimulated the search for other definitions. In 2016, adult practitioners chose a definition based on life-threatening organ dysfunction rather than the SIRS criteria.^(6)^The Sepsis-3 Task Force analyzed data from nearly 150,000 adult patients in two high-income countries and concluded that sepsis should be defined by the presence of life-threatening organ dysfunction caused by a dysregulated host response to infections and identified sepsis using an increase in the Sequential Organ Failure Assessment (SOFA) score of at least 2 points in patients with suspected infection. These criteria, however, were not developed with pediatric data and have not been validated in children; therefore, they were not extrapolatable or adapted for children.

New criteria for defining pediatric sepsis have been eagerly awaited for years, and a task force led by the SCCM, comprising 35 experts from six continents, has worked diligently since 2019 to finalize these criteria. Published in January 2024, the Phoenix Sepsis Score (PSS) criteria were developed through 3 distinct methodologies that progressively built on and dove-tailed together. Data from a global survey about sepsis definition perceptions of 2,835 clinicians^(7)^ and a systematic review and meta-analysis^(5)^ helped guide the design of the last step. Finally, a data-driven derivation and validation study was conducted as a multicenter retrospective cohort study^(2)^ and concluded with a modified Delphi consensus process where all members of the task force reviewed, discussed, and voted upon the criteria. The dataset is to be commended for its robustness, as it included 172,984 pediatric encounters from 10 health systems in both high-income countries (HICs) and low-middle income countries (LMICs).^(2)^

The final conceptual new definition for pediatric sepsis is “an infection with life-threatening organ dysfunction”, which includes a four-organ system model—the PSS—with respiratory, cardiovascular, coagulation, and neurological variables. Sepsis is identified by a PSS of at least 2 points in children with suspected infection, and septic shock is defined as children with sepsis with at least 1 cardiovascular point in PSS. The variables used, such as PaO_2_/FiO_2_ or SpO_2_/FiO_2_ for respiratory dysfunction, the Glasgow Coma Scale score for neurologic dysfunction and the use of amines for cardiovascular dysfunction, are indubitable. The authors, however, note that the PSS includes lower mean arterial pressure cutoff values than most currently used pediatric blood pressure values,^(8,9)^ which is surprising because of the possibility of not scoring this parameter for borderline hypotensive children. Additionally, the PSS does not include the capillary refill time, a universally available physical examination finding, in the criteria. Prolonged capillary refill time reflects damage to the microcirculation^(10)^ and is a surrogate for laboratory values such as lactate, which may not be available in many limited-resource settings. Future prospective studies must evaluate the exclusion of this important physical finding and its impact on septic patient identification and outcomes.

However, we do note that since these findings were derived and validated using data from high- and limited-resource settings, the new criteria should improve the diagnosis of pediatric sepsis and septic shock globally compared to the existing IPSCC criteria. The PSS criteria should have a greater positive predictive value and a similar or better sensitivity for identifying life-threatening organ dysfunction. These improvements are also essential for enriching clinical trial entry criteria, antibiotic stewardship, and more reliable global data on sepsis-related mortality.

Nineteen years after the historic creation of the IPSCC criteria, a new task force for pediatric sepsis definitions identified both the gaps in the original criteria and a potential solution. This bold effort must be commended, and the rest of the community must critically evaluate the Phoenix criteria on several levels. The Phoenix criteria outperformed the IPSCC criteria, which has been the sole pediatric sepsis definition to date. However, despite incorporating data from both HICs and LMICs for development and validation, the external validity of the PSS will be confirmed only once validated screening tools are studied and developed. Regrettably, there are few studies on sepsis screening tools in both HIC and LMIC settings, hindering the selection of a single tool or the determination of criteria to identify patients at risk for sepsis.^(11,12)^The 2020 guidelines from the Pediatric Surviving Sepsis Campaign advocate for health care institutions to adapt systematic screening for acutely unwell children, aiming to more promptly identify septic shock and other sepsis-related organ dysfunctions.^(13)^Thus, establishing the fundamental requirements for a sepsis screening tool that ensures consistency in the variables used across multiple settings and institutions is an essential next step. The prerequisites can then be tailored to the specificities of each scenario (e.g., electronic vs. nonelectronic; frequency of re-evaluation; parental concerns, health-care provider intuition). Once sepsis screening tools meeting established prerequisites are available, assessing the external validity and performance of the PSS will be feasible. However, the lack of uniformity among screening tools poses challenges in this assessment.

What comes next? First, the PSS must be further validated globally and prospectively in various resource settings and among a broad range of patients ranging from previously healthy children to those with malnutrition or chronic illness. Second, the new score must be adapted by medical systems that create and implement high-quality screening tools with the new Phoenix criteria to allow early identification of sepsis patients at the institutional level, and front-line providers/researchers must adopt this future method of sepsis diagnosis. Third, a follow-up survey should be conducted broadly to identify the benefits, limitations, and challenges of using the new criteria. Fourth, and most importantly, will the Phoenix criteria improve patient outcomes? This new conceptual framework has simplified the diagnosis to rapidly identify life-threatening organ dysfunction due to infection, but this simplicity should not limit the diagnosis or delay treatment of patients with early sepsis or less severe infection. The success of this paradigm shift in caring for pediatric sepsis patients in the critical care community will lie in continued validation of the criteria, in creating systematic changes for standardized diagnosis, in identifying limitations and challenges, and, finally, in demonstrating improvements in patient outcomes.
