# Supplementary material for: New Phoenix criteria for pediatric sepsis and septic shock: the strengths and the future of a comprehensive perspective
Source: Crit Care Sci. 2024 Jul 1;36:e20240058en. doi: 10.62675/2965-2774.20240058-en (PMC11239205; doi:10.62675/2965-2774.20240058-en)
Supplement: Translation

# Translation: Novos critérios Phoenix para sepse e choque séptico em pediatria: os pontos fortes e o futuro de uma perspectiva abrangente

**DOI:** 10.62675/2965-2774.20240058-pt

**Authors:** Vanessa Soares Lanziotti, Andrea Ventura, Saraswati Kache, Jaime Fernández-Sarmiento

**Affiliations:** 1 Brasil Unidade de Terapia Intensiva Pediátrica e Divisão de Ensino e Pesquisa, Instituto de Puericultura e Pediatria Martagão Gesteira - Rio de Janeiro (RJ), Brasil.; 2 Brasil Departamento de Pediatria, Universidade de São Paulo - São Paulo (SP), Brasil.; 3 Estados Unidos Divisão de Medicina Intensiva, Departamento de Pediatria, Lucile Packard Children’s Hospital Stanford - Califórnia, Estados Unidos.; 4 Colômbia Departamento de Medicina Intensiva e Pediatria, Fundación Cardioinfantil, Instituto de Cardiologia, Universidad de la Sabana - Bogotá, Colômbia.

Na mitologia grega, a ave fênix simboliza a vida que vence a morte e a força que acompanha a transformação. Assim, Phoenix é um nome apropriado para o novo *Pediatric Sepsis Score* devido à referência mitológica e ao local onde ele foi apresentado pela primeira vez (*Society of Critical Care Medicine* - SCCM - Conferência em Phoenix, Arizona).^(1)^ Os critérios de Phoenix para sepse e choque séptico pediátricos (*Phoenix Pediatric Sepsis* - PPS) destinam-se a identificar crianças (de 1 mês a < 18 anos) com disfunção orgânica com risco de morte devido à infecção, e o escore foi desenvolvido com base em mais de 3 milhões de registros eletrônicos pediátricos em sistema de saúde,^(2)^ o que é uma conquista notável entre os estudos de sepse pediátrica e de adultos. Os critérios anteriores de sepse pediátrica foram publicados em 2005 pela *International Pediatric Sepsis Consensus Conference* (IPSCC), e a sepse foi definida como uma infecção suspeita ou confirmada na presença da síndrome de resposta inflamatória sistêmica (SIRS) (Figura 1).^(3)^ Embora esses critérios sejam amplamente utilizados na prática diária, foram identificadas limitações a essa definição desde seu início.^(4)^ Entre as limitações específicas que causam preocupação, estão a falta de consideração de um contexto global, o que leva a desafios na aplicabilidade desses critérios em ambientes com recursos limitados, onde se encontra a maior incidência de sepse; a variabilidade na aplicação à beira do leito, o que leva a atrasos no diagnóstico do paciente; e a incapacidade de identificar os pacientes com maior risco de desfechos desfavoráveis.^(5)^

**Figura 1:** Comparação dos critérios da *International Pediatric Sepsis Consensus Conference* com os critérios da *Phoenix Pediatric Sepsis* em termos de objetivos, definições, critérios, classificação e metodologia. * Pesquisa internacional, revisão sistemática/metanálise e um estudo de coorte; † a definição de disfunção de órgãos cardiovasculares dos critérios da *International Pediatric Sepsis Consensus Conference* é a seguinte: 40mL/kg ou mais de fluido isotônico em 1 hora, hipotensão ou necessidade de fluido vasoativo ou pelo menos 2 dos seguintes itens: acidose metabólica inexplicada, lactato arterial > 2 vezes o limite superior do normal, oligúria, tempo de enchimento capilar prolongado ou diferença de temperatura entre o núcleo e a periferia; ‡ a definição de disfunção de órgãos cardiovasculares do Phoenix inclui hipotensão grave para a idade, valor de lactato no sangue venoso ou arterial superior a 5mmol/L (> 45,05mg/dL), ou a necessidade de medicação vasoativa. SIRS - síndrome de resposta inflamatória sistêmica.

Essas limitações do IPSCC estimularam a busca por outras definições. Em 2016, intensivistas de pacientes adultos optaram por uma definição baseada na disfunção orgânica com risco de vida ao invés dos critérios de SIRS.^(6)^ A força-tarefa responsável pelo Sepsis-3 analisou dados de quase 150 mil pacientes adultos em dois países de alta renda e concluiu que a sepse deve ser definida pela presença de disfunção orgânica com risco de vida causada por uma resposta desregulada do hospedeiro a infecções, além de ter identificado a sepse por meio do aumento na pontuação do *Sequential Organ Failure Assessment* (SOFA) de pelo menos dois pontos em pacientes com suspeita de infecção. Esses critérios, no entanto, não foram desenvolvidos com dados pediátricos e nem validados em crianças; portanto, não eram extrapoláveis e nem adaptados para crianças.

Novos critérios para definir sepse pediátrica são aguardados com ansiedade há anos, e uma força-tarefa liderada pela SCCM, composta de 35 especialistas de seis continentes, tem trabalhado diligentemente desde 2019 para finalizar esses critérios. Publicados em janeiro de 2024, os critérios de Phoenix (*Phoenix Sepsis Score* - PSS) foram desenvolvidos por meio de três metodologias distintas que foram progressivamente construídas e articuladas. Os dados de uma pesquisa global sobre as percepções da definição de sepse de 2.835 médicos^(7)^ e uma revisão sistemática e metanálise^(5)^ ajudaram a orientar o desenho da última etapa. Por fim, um estudo de derivação e validação orientado por dados foi conduzido como um coorte retrospectivo multicêntrico^(2)^ e concluído com um processo de consenso Delphi modificado, no qual todos os membros da força-tarefa revisaram, discutiram e votaram nos critérios. O conjunto de dados deve ser elogiado por sua robustez, pois incluiu 172.984 encontros pediátricos de 10 sistemas de saúde em países de alta (HICs - *high-income countries*) e baixa e média renda (LMICs - *low-middle income countries*).^(2)^

A nova definição conceitual final para sepse pediátrica é “uma infecção com disfunção orgânica com risco de morte”, que inclui um modelo de sistema de quatro órgãos – o PSS - com variáveis respiratórias, cardiovasculares, de coagulação e neurológicas. A sepse é identificada por um PSS de pelo menos dois pontos em crianças com suspeita de infecção, e o choque séptico é definido como crianças com sepse com pelo menos um ponto cardiovascular no PSS. As variáveis utilizadas, como pressão parcial de oxigênio/fração inspirada de oxigênio (PaO_2_/FiO_2_) ou saturação de oxigênio (SpO_2_)/FiO_2_ para disfunção respiratória, o escore da escala de coma de Glasgow para disfunção neurológica e o uso de aminas para disfunção cardiovascular, são indubitáveis. Os autores, no entanto, observam que o PSS inclui valores de corte de pressão arterial média mais baixos do que a maioria dos valores de pressão arterial pediátrica usados atualmente,^(8,9)^ o que é surpreendente devido à possibilidade de não pontuar esse parâmetro em crianças hipotensas limítrofes. Além disso, o PSS não inclui nos critérios o tempo de enchimento capilar, um achado de exame físico universalmente disponível. O tempo de enchimento capilar prolongado reflete danos à microcirculação^(10)^ e é um substituto para valores laboratoriais, como o lactato, que pode não estar disponível em muitos locais com recursos limitados. Estudos prospectivos futuros devem avaliar a exclusão desse importante achado físico e seu impacto na identificação e nos desfechos de pacientes sépticos.

No entanto, observamos que, como esses achados foram derivados e validados usando dados de locais com recursos altos e limitados, os novos critérios devem melhorar o diagnóstico da sepse pediátrica e do choque séptico globalmente em comparação com os critérios IPSCC existentes. Os critérios de Phoenix têm valor preditivo positivo maior e uma sensibilidade semelhante ou melhor para identificar a disfunção de órgãos com risco de vida. Essas melhorias também são essenciais para aprimorar os critérios de entrada em estudos clínicos, o uso racional de antibióticos e dados globais mais confiáveis sobre a mortalidade relacionada à sepse.

Depois de 19 anos da criação histórica dos critérios do IPSCC, uma nova força-tarefa para definições de sepse pediátrica identificou as lacunas nos critérios originais e uma possível solução. Esse esforço ousado deve ser elogiado, e o restante da comunidade deve avaliar criticamente os critérios de Phoenix em vários níveis. Os critérios de Phoenix superaram os critérios do IPSCC, que tem sido a única definição de sepse pediátrica até o momento. Entretanto, apesar de incorporar dados de HICs e LMICs para desenvolvimento e validação, a validade externa do PSS será confirmada somente quando ferramentas de triagem validadas forem estudadas e desenvolvidas. Lamentavelmente, há poucos estudos sobre ferramentas de triagem de sepse em ambientes de HIC e LMIC, o que dificulta a seleção de uma única ferramenta ou a determinação de critérios para identificar pacientes em risco de sepse.^(11,12)^ As diretrizes de 2020 da *Pediatric Surviving Sepsis Campaign* defendem que as instituições de saúde adaptem a triagem sistemática para crianças com mal-estar agudo, com o objetivo de identificar mais prontamente o choque séptico e outras disfunções orgânicas relacionadas à sepse.^(13)^ Portanto, estabelecer os requisitos fundamentais para uma ferramenta de triagem de sepse que garanta a consistência das variáveis usadas em vários ambientes e instituições é uma próxima etapa essencial. Os pré-requisitos podem então ser adaptados às especificidades de cada cenário (por exemplo: eletrônico *versus* não eletrônico; frequência de reavaliação; preocupações dos pais, intuição do profissional de saúde). Quando as ferramentas de triagem de sepse que atendem aos pré-requisitos estabelecidos estiverem disponíveis, será possível avaliar a validade externa e o desempenho do PSS. Entretanto, a falta de uniformidade entre as ferramentas de triagem apresenta desafios para essa avaliação.

O que vem a seguir? Primeiro, o PSS deve ser validado de forma ainda mais global e prospectivamente em várias configurações de recursos e entre uma ampla gama de pacientes, desde crianças previamente saudáveis até aquelas com desnutrição ou doença crônica. Em segundo lugar, a nova pontuação deve ser adaptada pelos sistemas médicos que criam e implementam ferramentas de triagem de alta qualidade com os novos critérios de Phoenix para permitir a identificação precoce de pacientes com sepse em nível institucional, e os provedores/pesquisadores da linha de frente devem adotar esse futuro método de diagnóstico de sepse. Em terceiro lugar, uma pesquisa de seguimento deve ser realizada de forma ampla para identificar os benefícios, as limitações e os desafios do uso dos novos critérios. Quarto, e mais importante, os critérios de Phoenix melhorarão os desfechos dos pacientes? Essa nova estrutura conceitual simplificou o diagnóstico para identificar rapidamente a disfunção de órgãos com risco de vida devido à infecção, mas essa simplificação não deve limitar o diagnóstico ou atrasar o tratamento de pacientes com sepse precoce ou infecção menos grave. O sucesso dessa mudança de paradigma no atendimento de pacientes pediátricos com sepse na comunidade de terapia intensiva estará na validação contínua dos critérios, na criação de mudanças sistemáticas para o diagnóstico padronizado, na identificação de limitações e desafios e, finalmente, na demonstração de melhorias nos desfechos dos pacientes.
